# Effects of ginsenoside Re on LPS-induced inflammatory mediators in BV2 microglial cells

**DOI:** 10.1186/1472-6882-12-196

**Published:** 2012-10-26

**Authors:** Kang-Woo Lee, So Young Jung, Sun-Mi Choi, Eun Jin Yang

**Affiliations:** 1 Department of Medical Research, Korea Institute of Oriental Medicine, 483 Expo-ro, Yuseong-gu, Daejeon, 305-811, Republic of Korea

**Keywords:** Ginsenoside-Re (G-Re), BV2 microglial cell, Neuroinflammation, Phospho-p38MAPK

## Abstract

**Background:**

Microglial activation plays an important role in neurodegenerative diseases by producing several pro-inflammatory enzymes and pro-inflammatory cytokines. Lipopolysaccharide (LPS)-induced inflammation leads to the activation of microglial cells in the central nervous system (CNS) and is associated with the pathological mechanisms of neurodegenerative diseases, including PD, AD, and ALS. Ginseng is a natural antioxidant used in herbal medicine and contains ginsenosides (Rb1, Rg1, Rg3, Re, and Rd), which have anti-neoplastic and anti-stress properties.

This study demonstrates the involvement of the anti-inflammatory signaling pathway, ginsenoside-Re (G-Re), which is one of the ginsenosides mediated by LPS-induced neuroinflammation in BV2 microglial cells.

**Methods:**

BV2 microglial cells were pretreated with 2 μg/ml G-Re and stimulated with 1 μg/ml LPS to induce neuroinflammation. To investigate the effect of G-Re on LPS-induced cell signaling, we performed western blotting and immunofluorescence using specific antibodies, such as phospho-p38, COX2, and iNOS.

**Results:**

Pretreatment with 2 μg/ml G-Re was neuroprotective against 1 μg/ml LPS-treated microglial cells. The neuroprotective events induced by G-Re treatment in neuroinflammation occurred via the phospho-p38, iNOS, and COX2 signaling pathways in BV2 cells.

**Conclusion:**

Taken together, we suggest that G-Re exerts a beneficial effect on neuroinflammatory events in neurodegenerative diseases.

## Background

Microglial cells, which are the major immune cells in the brain, play a pivotal role in the innate immune response in the central nervous system (CNS) [1]. The activation of microglia releases various neurotrophic factors that support neuronal cell survival, in addition to neurotoxic factors and pro-inflammatory cytokines [2]. Acute activation causes several autoimmune responses to neuronal death and brain injury. The activation of microglia in the CNS is associated with the pathogenesis of a variety of neurodegenerative diseases, such as Multiple Sclerosis (MS), Alzheimer’s disease (AD), Huntington’s disease (HD), and Parkinson’s disease (PD) [3]. The overactivation of microglia and the consequent release of pro-inflammatory and cytotoxic factors, including the tumor necrosis factor-(TNF-α), inducible nitric oxide synthase (iNOS), and cyclooxygenase 2 (COX2), contribute to neurodegenerative processes [4].

A recent study has reported that the activation of microglia can trigger neurotoxicity via the production of pro-inflammatory and cytotoxic factors in neuronal cell lines treated with lipopolysaccharide (LPS), β-amyloid, glutamate, and arachidonate [5]. LPS, which is a bacterial endotoxin, induces inflammation, tissue damage, infection, and inflammatory responses. LPS is widely used to activate macrophage-like cells and to simulate infection. LPS-treated BV2 cells express CD14, interleukin-6 (IL-6), TNF-α, resulting in increased levels of iNOS [6-8]. LPS stimulates nuclear factor-κB (NF-κB), cyclic AMP-responsive element-binding protein (CREB) and the mitogen-activated protein kinase (MAPKs) family, including extracellular signal-regulated kinases (ERKs), c-Jun N-terminal kinase (JNK), and p38 MAPK [9], which have been implicated in the release of immune-related cytotoxic factors such as iNOS, COX2, and pro-inflammatory cytokines [4,10]. Thus, the control of microglial activation has been suggested as a promising therapeutic target to combat neurodegenerative diseases.

Ginseng, one of the most ancient herbs used in traditional Chinese medicine, exhibits anti-inflammatory properties. Active constituents with curable features can be found in most ginseng species, including ginsenosides, polysaccharides, peptides, polyacetylenic alcohols, and fatty acids. There are two major categories of ginsenosides; protopanaxadiols (PPD, e.g., Ra, Rb, Rc, Rd, Rg3, Rh2) and protopanaxatriols (PPT, e.g., Re, Rf, Rg1, Rg2, Rh1). Ginsenoside Rg1 (Rg1), one of the saponin components of ginseng, has been widely reported for its neuroprotective effects on the CNS. Ginsenoside-Re (G-Re) exhibits anti-oxidative capabilities in addition to its neuroprotective activities, and it also demonstrates anti-hyperlipidemic and immunomodulatory therapeutic properties [11,12], and anti-inflammatory effects [13]. Recent studies have reported that the major active ingredients, including ginsenosides, exert anti-oxidant and anti-inflammatory effects [13-15]. Wu et al. showed that G-Re has anti-inflammatory effect by inhibition of nitric oxide (NO) formation and NF-κB signaling in the LPS-induced microglial cell [13].

Several studies have reported the neuroprotective effects of Rg1 or its metabolites, but not of Re. Thus, we investigated the anti-inflammatory effects of G-Re on LPS-stimulated microglial BV2 cells, and we provide insight on its molecular mechanism. We provide further evidence for the anti-inflammatory potential of G-Re *in vitro* and the involvement of the signaling molecules, phospho-p38, iNOS, and COX2. These results provide a scientific basis for further investigation of G-Re as therapeutic agent for the treatment of neuroinflammatory diseases.

## Methods

### Cell culture

The immortalized BV2 murine microglial cell line was provided by Dr. Sang-Myun Park (Aju University, Republic of Korea) and grown in Dulbecco’s modified Eagle’s medium (DMEM) supplemented with 10% FBS (fetal bovine serum), 100 U/ml penicillin, and 100 μg/ml streptomycin at 37°C in an atmosphere of 5% CO_2_ in air**.** In all of the experiments, BV2 cells were incubated in the presence or absence of 2 μg/ml of G-Re before the addition of LPS (Enzo, Farmingdale, NY, USA) to the culture media.

### Cell viability assay

Cell viability was assessed by an MTT (3-[4,5-dimethyl-thiazol-2-yl]-2,5-diphenyltetrazolium bromide) reduction assay, as described previously [16]. This assay is based on the ability of active mitochondrial dehydrogenase to convert dissolved MTT into water-insoluble purple formazan crystals. BV2 cells were plated on 96-well plates (2 × 10^4^ cells/well). After 24 h of cell seeding, the BV2 cells were treated with the indicated concentrations of G-Re for 24 h prior to 1 μg/ml of LPS treatment for an additional 24 h. Briefly, MTT was added to each well at a final concentration of 0.5 mg/ml, and the plates were incubated for 1 h at 37°C. After removal of the culture medium, DMSO was added, and the plates were shaken for 10 min to solubilize the formazan reaction product. The absorbance at 570 nm was measured using a microplate reader (Bio-rad, xMark). The absorbance at 570 nm was expressed as the percent of the relative untreated control BV2 cells and reported as the mean.

### Western blot

After treatment with or without 1 μg/ml LPS in the presence of 2 μg/ml G-Re, the cells were washed with ice-cold PBS and lysed with RIPA lysis buffer containing 50 mM Tris–HCl pH 7.4, 1% NP-40, 0.1% SDS, 150 mM NaCl, and the Complete Mini Protease Inhibitor Cocktail (Roche, Basel, Switzerland). The protein concentration was measured with a BCA Protein Assay Kit (Pierce, IL, USA). Extracted samples (20 μg total proteins per lane) were separated by 10% SDS-polyacrylamide gel electrophoresis (SDS-PAGE) and then transferred onto nitrocellulose membranes (Whatman, Dassel, Germany). The membranes were incubated with 5% skim milk to block nonspecific protein binding and incubated with primary antibodies for p-p38 (1:1000, Cell Signaling), p-JNK (1:1000, Cell Signaling), *α*-tubulin (1:5000, Abcam), iNOS (1:1000, BD Pharmingen), and COX-2 (1:1000, BD Pharmingen) in 5% skim milk overnight. After washing 3 times with TBS-T (1 M Tris–HCl pH 7.5, 1.5 M NaCl, 0.5% tween-20), the membranes were hybridized with horseradish peroxidase-conjugated secondary antibodies for 1 h. Then, the membranes were washed with TBS-T, and the specific immunoreactive protein bands were detected using the SuperSignal West Femto Chemiluminescent Substrate (Pierce, IL, USA) or enhanced with chemiluminescence reagents (Amersham Pharmacia, NJ, USA). *α*-tubulin was used as an internal control to normalize for protein loading. Protein bands were detected and analyzed using a FusionSL4-imaging system, and quantification of the immunoblotting bands was performed with the Bioprofil (Bio-1D version 15.01, viber Lourmart).

### Immunofluorescence

For immunofluorescence staining, the cells were washed with PBS, and then fixed with 4% paraformaldehyde for 15 min and permeabilized with 0.5% Triton X-100 in PBS for 10 min. Fixed cells were then blocked with 5% BSA in PBS-T (0.1% Triton X-100 in PBS) to reduce nonspecific immune reactivity, and the cells were incubated with primary antibodies overnight at 4°C. After washing 3 times with PBS-T, the cells were incubated with secondary antibodies conjugated to FITC or rhodamine. Stained cells were mounted with fluorescence mounting medium with DAPI (Vector laboratories, CA, USA). The fluorescently stained cells were then examined using a microscope (Olympus, BX51). The number of fluorescently stained cells was counted in each of the three randomly chosen fields using the NIH *Image J* program (version 1.46j).

### Data analysis

Data are expressed as the mean ± S.E.M. Comparisons were evaluated by one-way analysis of variance (ANOVA) with Prism software. Values that were significantly different from the relative controls are indicated with an asterisk when p < 0.05.

## Results

### Ginsenoside-Re prevents LPS-induced microglial cell death in BV2 microglial cells

**Figure 1:**
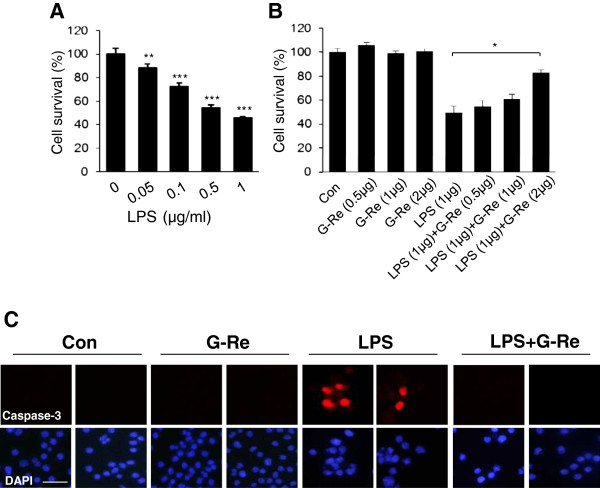
**Effects of ginsenoside-Re on viability in LPS-induced BV2 microglial cells. **(**A**) G-Re inhibits LPS-induced cell death in BV2 microglial cells. BV2 microglial cells were treated with indicated doses (0, 0.05, 0.1, 0.5 and 1 μg/ml) of LPS for 24 h, and cell viability was measured by an MTT assay. The values shown are the mean ± S.E.M. of data obtained from three independent experiments. n=15, * p < 0.05, ** p < 0.01. (**B**) Effects of G-Re on LPS-induced microglial cell death. BV2 cells were pretreated for 24 h with 2 μg/ml G-Re, and then stimulated with 1 μg/ml LPS for 24 h in the presence or absence of G-Re. The rates of cell survival were measured by a MTT assay at 570 nm. The values shown are the mean ± S.E.M. of data obtained from three independent experiments. (**C**) BV2 cells were pretreated with 2 μg/ml G-Re for 24 h and then stimulated to 1 μg/ml LPS for 24 h in the presence or absence of 2 μg/ml G-Re. Cells were visualized using immunofluorescence microscopy with antibodies against active caspase-3. The values shown are the mean ± S.E.M. of data obtained from three independent experiments. (bars: 50 μm.) * p < 0.05.

To examine the viability of BV2 microglia after LPS treatment, we incubated BV2 microglial cells with LPS (1 μg/ml) at the indicated doses for 24 h. Our results showed that LPS decreased cell survival in a dose-dependent manner (Figure 1A). Compared to vehicles, 1 μg/ml LPS treatment of BV2 cells resulted in a decrease in cell viability by 54%. To investigate whether G-Re attenuated LPS-induced microglial cell death, BV2 microglial cells were treated with G-Re plus LPS. After pretreatment of G-Re (0.5, 1 and 2 μg/ml) for 24 h, BV2 cells were treated with LPS for 24 h in the presence or absence of G-Re. Treatment of LPS alone markedly decreased cell survival; however, pretreatment with G-Re reduced this decrease of cell survival by 84% at a dose of 2 μg/ml G-Re (Figure 1B). In addition, immunocytochemical analysis showed that the levels of active caspase-3, a key enzyme that regulates cell apoptosis, were increased at 24 h after LPS treatment. Pretreatment with G-Re for 24 h inhibited the upregulation of activated caspase-3 (Figure 1C). These results indicate that G-Re exhibits a protective role against LPS-induced microglial cell death.

### Ginsenosides-Re attenuates LPS-induced activation of p38MAPK in BV2 microglial cells

**Figure 2:**
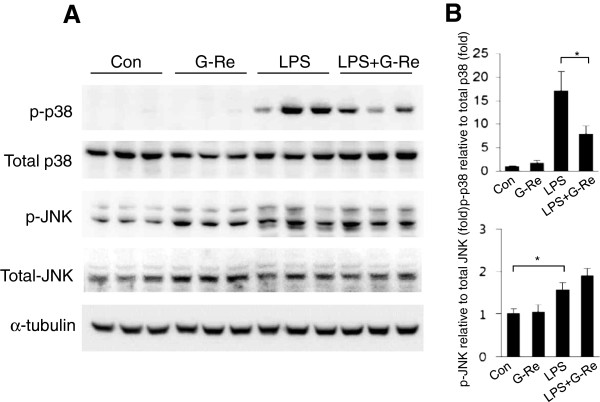
**Ginsenoside-Re inhibits LPS-induced phosphorylation of p38MAPK in BV2 microglial cells. **(**A**) The level of phosphorylated p38MAPK was highly increased by LPS alone and was diminished by G-Re. BV2 cells were pretreated with 2 μg/ml G-Re for 1 h and then stimulated with 1 μg/ml LPS for 15 min in the presence or absence of 2 μg/ml G-Re. Western blots were performed with specific antibodies, including the phosphorylated form of p38 and JNK. Total p38, JNK and α-tubulin were used as loading controls for the cell lysates. The values shown are the mean ± S.E.M. of data obtained from three independent experiments. * p < 0.05, ** p < 0.01. (**B**) Quantification of A. No differences were found with the G-Re alone treatments. * p < 0.05, ** p < 0.01.

Because the MAPK family is known to be a key player in LPS-induced cell signaling, we examined whether G-Re decreases the phosphorylation of the MAPK family proteins, JNK and p38MAPK. To do this, BV2 microglial cells were treated with 1 μg/ml LPS in the presence or absence of 2 μg/ml G-Re. A significant increase in JNK and p38 phosphorylation was observed as early as 15 min after LPS treatment (Figure 2). However, pretreatment with G-Re attenuated LPS-induced phosphorylation of p38 in the presence of G-Re and not JNK. Moreover, the total protein levels of JNK and p38 were unchanged (Figure 2). These results suggest that the G-Re-mediated attenuation of cell death is associated with downregulation of the p38MAPK signaling pathway.

### Ginsenosides-Re attenuates the protein expression of LPS-induced pro-inflammatory mediators in BV2 microglial cells

**Figure 3:**
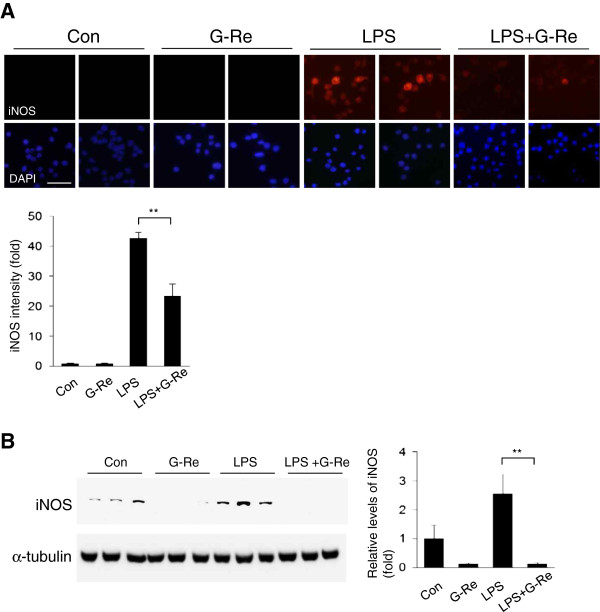
**Ginsenoside-Re attenuates the expression levels of iNOS proteins in LPS-induced BV2 microglial cells. **(**A**) BV2 cells were pretreated with 2 μg/ml of G-Re for 1 h, and then stimulated with 1 μg/ml LPS for 18 h in the presence or absence of 2 μg/ml G-Re. Cells were visualized using immunofluorescence microscopy with antibody against iNOS. Quantification of iNOS expression density by *Image J* is shown on the right. (n = 5 for each of the four groups.) (bars: 50 μm.) * p < 0.05, ** p < 0.01. (**B**) Western blots were performed using an antibody against iNOS. The loading control for the cell lysates was determined by re-probing the membranes with α-tubulin antibody. The values shown are the mean ± S.E.M. of data obtained from three independent experiments. * p < 0.05, ** p < 0.01.

**Figure 4:**
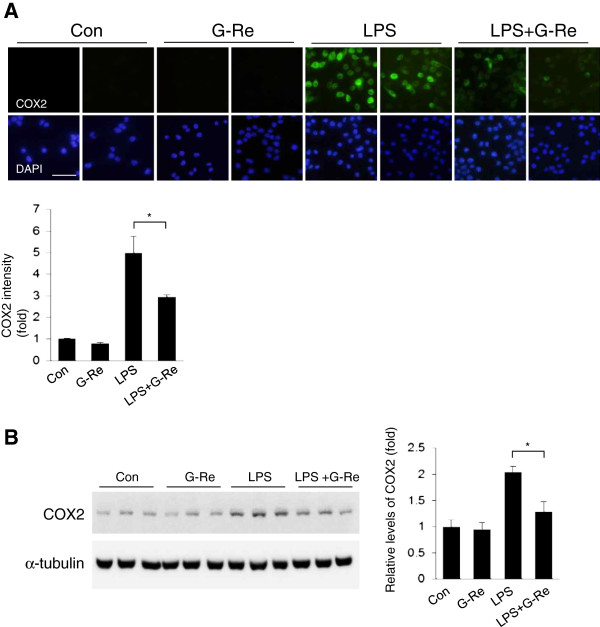
**Ginsenosides-Re attenuates the expression levels of COX2 proteins in LPS-induced BV2 microglial cells. **(**A**) BV2 cells were pretreated with 2 μg/ml of G-Re for 1 h, and then stimulated with 1 μg/ml LPS for 18 h in the presence or absence of 2 μg/ml G-Re. Cells were visualized using immunofluorescence microscopy with antibody for COX2. Quantification of COX2 expression density by *Image J* is shown on the right (n = 5 for each of the four groups.) (bars: 50 μm.) * p < 0.05, ** p < 0.01. (**B**) Western blots were performed using an antibody against COX2. The loading control for the cell lysates was determined by re-probing the membranes with α-tubulin antibody. The values shown are the mean ± S.E.M. of data obtained from three independent experiments. * p < 0.05, ** p < 0.01.

To investigate the effect of G-Re on LPS-induced microglial activation, BV2 microglial cells were treated with 1 μg/ml LPS for 18 h. LPS highly increased the protein levels of iNOS and COX2 (Figure 3 and 4) in BV2 microglial cells. Western blot analysis showed that treatment with 2 μg/ml G-Re markedly inhibited iNOS and COX2 protein levels compared with LPS alone (Figure 3B and 4B). As shown in Figure 3A and 4A, we confirmed the inhibitory effect of G-Re on LPS-induced BV2 microglial cells by immunofluorescence staining. Stimulation of BV2 microglial cells with 1 μg/ml LPS resulted in a notable increase in the protein expression levels of iNOS and COX2. Compared with LPS treatment alone, these expression levels were decreased by G-Re treatment. These findings indicated that G-Re inhibits LPS-induced pro-inflammatory protein expression in BV2 microglial cells.

## Discussion

The present study demonstrates that G-Re inhibits LPS-induced pro-inflammatory mediators in BV2 microglial cells through the blockade of the p38MAPK signaling pathway. Stimulation of microglial cells with LPS induced the activation of p38, which subsequently led to the upregulation of iNOS and COX2 expression. G-Re also significantly suppressed the LPS-induced expression of iNOS and COX2 in BV2 microglial cells. Thus, our data support that p38MAPK is important for microglial cell death and pro-inflammatory cytokine upregulation in response to LPS and may be a therapeutic target for neuroinflammatory diseases, where overproduction of pro-inflammatory cytokines has been implicated in disease progression. G-Re is one promising therapeutic approach for the treatment of neuroinflammatory diseases.

Ginseng is a traditional herbal agent that has been used in traditional Chinese medicine. Ginseng contains a mixture of 30 heterogeneous glycosidal saponins, which are also known as ginsenosides. Ginsenosides including Rb1, Rg1, Rg3, Re, and Rd and their biomodulating and immunomodulating functions in the immune system and CNS have been examined in clinical and animal studies [17,18]. Commonly studied ginsenosides such as Rb1, Rg1, Rg3, Re, and Rd exhibit vasorelaxation, anti-oxidation, and anti-cancer functions. In particular, G-Re, a compound derived from Panax ginseng, improved anti-diabetic effects by suppression of phospho-JNK and NF-κB in diabetic animals [19]. In addition, G-Re treatment improved cognitive impairment and helpless behaviors by regulation of the noradrenergic system in animal models [20]. However, the molecular mechanisms of G-Re remain unknown.

Microglial cells are generally considered to be the immune cells of the CNS. Microglial cells respond to neuronal injury or damage with microglial activation. Activated microglia produce large amounts of harmful neurotoxic factors through the excess production of cytotoxic factors, such as superoxide radicals, nitric oxide [21], TNF-α and interleukin-1β [22]. p38MAPK is a serine/threonine MAPK that is activated by a wide range of environmental stressors and cytokines to induce inflammation. p38MAPK is an important regulator of pro-inflammatory cytokines such as iNOS and COX2 [23]. In several studies, phosphorylation/activation of p38MAPK was increased by LPS treatment and, subsequently, the p38 inhibitor suppressed LPS-induced pro-inflammatory cytokine upregulation in BV2 microglial cells [23]. This study showed that G-Re reduced LPS-induced neuroinflammation by inhibition of the p38-activating signaling pathway in BV2 microglial cells (Figure 2). This result suggests that G-Re has a neuroprotective role against neuroinflammation in the CNS. However, our data indicate that LPS-induced JNK activation was not decreased by G-Re (Figure 2). Several researchers found that ginsenoside Re, ginsenoside Rb1 and ginsenoside Rg1 has a beneficial effect in LPS-activated microglial cell through inhibition of nitric oxide (NO) formation and NF-κB signaling [13,24,25]. Moreover, G-Re has a neuroprotective effect against LPS-induced cell death (Figure 1).

We treated with 0.5–100 μg/ml of G-Re to determine cell toxicity and found that G-Re treatment did not induce cell toxicity in BV2 cells, in accordance with a previous paper. In addition, we found that treatment with 2 μg/ml of G-Re inhibited LPS-induced cell death, as well as the activation of p38MAPK and activated caspase-3 by LPS treatment in BV2 cells, as shown in Figure 1C and 2A. However, we expect that a high dose (more than 2 μg/ml) of G-Re could attenuate LPS-induced cell death and change the expression of other proteins to a greater extent than in the present study. According to Wu et al.’s paper [13], they used 0.1–100 μM of G-Re. However, the effect of high-dose G-Re seems to have been similar to that of the low dose in nitric oxide or TNF-α production. In addition, the only high dose of G-Re treatment inhibited the pJNK and pIκBα expression. Therefore, we suggest that the effect of G-Re treatment will be increased at a high dose, depending on the target proteins and cell lines.

Previous studies have reported the development of hydrogen peroxide [26,27], glutamate [28], MPP+ [29] and β-amyloid-induced cell death [30]; however, the mechanism by which LPS leads to microglial toxicity remains obscure. In addition, it is noteworthy that G-Re has a protective effect with a markedly attenuated microglial response of caspase 3 activation (Figure 1).

It has been shown that the reduction of pro-inflammatory mediators produced by microglia may attenuate the severity of neuronal damage. Many studies have demonstrated that some pro-inflammatory cytokines and their reaction products are involved in chronic inflammatory disease [31,32]. COX2, an inducible isoform of COX, is upregulated in inflammation. Moreover, inducible nitric oxide synthase (iNOS) is associated with inflammation, and its reaction product NO is involved in various diseases, such as AD, PD and ALS [33-35]. Consistent with other researchers’ previous data regarding the signaling pathways mediated by microglial activation, our data indicate that G-Re exhibits anti-inflammatory effects, as shown in Figure 3 and 4.

## Conclusions

This study presented that the involvement of the anti-inflammatory signaling pathway by G-Re which is one of the ginsenosides mediated by LPS-induced neuroinflammation in BV2 microglial cells. Pretreatment with 2 μg/ml G-Re was neuroprotective against 1 μg/ml LPS-treated microglial cells. The neuroprotective events induced by G-Re treatment in neuroinflammation occurred via the phospho-p38, iNOS, and COX2 signaling pathways in BV2 cells. Taken together, the above *in vitro* findings suggest that G-Re mitigates neuroinflammatory events. Furthermore, there is a need to evaluate the effect of G-Re on toxin-induced or genetically engineered neuroinflammatory disease animal models. We therefore believe that inhibition of pro-inflammatory mediators may provide a new therapeutic approach for treatment of neuroinflammatory disease. In conclusion, our data provide evidence that G-Re attenuates LPS-induced microglial toxicity and pro-inflammatory activation, and these findings suggest that G-Re may serve as a potential therapeutic drug to delay neuroinflammatory progression.

## Competing interests

The authors declare no conflicts of interest.

## Authors’ contributions

EJY designed the experiments and analyzed the data as well as edited the manuscript. KWL and SYJ carried out biochemical experiments and performed statistical analyses. EJY, KWL, and SYJ wrote the manuscript. SMC provided some comments in writing the manuscript. All authors have read and approved the final manuscript.

## Pre-publication history

The pre-publication history for this paper can be accessed here:

http://www.biomedcentral.com/1472-6882/12/196/prepub
